# Factors influencing self-selected walking speed in fibrotic interstitial lung disease

**DOI:** 10.1038/s41598-021-91734-x

**Published:** 2021-06-14

**Authors:** Gabriela Fischer, Francisco B. de Queiroz, Danilo C. Berton, Pedro Schons, Henrique B. Oliveira, Marcelo Coertjens, Mathieu Gruet, Leonardo A. Peyré-Tartaruga

**Affiliations:** 1 Biomechanics Laboratory, Federal University of Santa Catarina, Florianópolis, SC Brazil; 2 Programa de Pós-Graduação Em Ciências Pneumológicas, Hospital de Clínicas de Porto Alegre/Universidade Federal Do Rio Grande Do Sul, Porto Alegre, RS Brazil; 3 Exercise Research Laboratory, Universidade Federal Do Rio Grande Do Sul, Rua Felizardo, 750, Porto Alegre, RS 90690-200 Brazil; 4 Programa de Pós-Graduação Em Ciências Do Movimento Humano, Universidade Federal Do Rio Grande Do Sul, Porto Alegre, RS Brazil; 5 Programa de Pós-Graduação Em Ciências Biomédicas, Universidade Federal Do Delta Do Parnaíba, Parnaíba, PI Brazil; 6 Laboratoire IAPS, Université de Toulon, 83041 Toulon, France

**Keywords:** Respiration, Respiratory tract diseases, Bioenergetics, Musculoskeletal system, Rehabilitation

## Abstract

This study aimed to investigate the walking economy and possible factors influencing self-selected walking speed (SSWS) in patients with fibrotic interstitial lung disease (ILD) compared to controls. In this study, 10 patients with ILD (mean age: 63.8 ± 9.2 years, forced expiratory volume in the first second: 56 ± 7% of predicted) and 10 healthy controls underwent resting pulmonary function tests, cardiopulmonary exercise, and submaximal treadmill walking tests at different speeds. The walking economy was assessed by calculating the cost-of-transport (CoT). Dynamic stability was assessed by stride-to-stride fluctuations using video recordings. Patients with ILD showed reduced peak oxygen uptake with a tachypneic breathing pattern and significant oxygen desaturation during exercise. The CoT did not differ between the groups (*p* = 0.680), but dyspnea and SpO_2_ were higher and lower, respectively, in patients with ILD at the same relative speeds. SSWS was reduced in ILD patients (2.6 ± 0.9 vs. 4.2 ± 0.4 km h^−1^ *p* = 0.001) and did not correspond to the energetically optimal walking speed. Dynamic stability was significantly lower in patients with ILD than in healthy controls, mainly at lower speeds. Patients with ILD presented a similar cost of transport compared to healthy controls; however, they chose lower SSWS despite higher walking energy expenditure. Although walking stability and dyspnea were negatively affected, these factors were not associated with the slower walking speed chosen by individuals with ILD.

## Introduction

Fibrotic interstitial lung disease (ILD) is a pathologically heterogeneous group of diseases^1–3^ characterized by impaired pulmonary diffusive potential and ventilatory mechanics^4,5^. In patients with fibrotic ILD, the progression of hypoxemia and dyspnea are associated with disability, reduced health-related quality of life, and reduced survival^6^.

As the disease progresses, breathlessness can impair the functional status of these patients, limiting daily activities^6^. In fact, functional capacity, assessed by field walking tests, is impaired and inversely related to the magnitude of dyspnea^7,8^. Interestingly, patients with reduced usual walking speed have significantly worse exercise performance and health status, despite similar lung function and fibrotic tomographic extension^7^. Although field walking tests are easy to perform and widely used in clinical practice^9^, they are unable to further our understanding of the mechanisms underlying reduced walking performance. Exercise test protocols assessing the energy economy profile and dynamic stability during walking speeds in daily life may provide more clarity^10^.

The cost of transport (CoT) is a movement-economy indicator that describes the mass-specific energy consumption required to travel a certain distance^11^. Individuals are more economical when they spend less energy to cover a given distance. Several studies^11–13^ have shown that the CoT depends on walking speed and that there is an optimal walking speed (OWS) for healthy young individuals (≈4 km h^−1^), where the energy expenditure is minimal and corresponds to the self-selected walking speed (SSWS). However, in certain conditions, the CoT is not minimized at SSWS, such as on rough terrain^14^ and while walking downhill^15^, where individuals prefer a more stable and costly gait pattern. In the elderly, SSWS matches the OWS, despite a higher CoT than that in the young individuals^16^. The lower economic walking performance in the elderly is supposed to be due an unstable walking pattern and sarcopenia/muscle weakness associated with aging^17,18^.

Studies on skeletal muscle dysfunction^19–21^ and impaired functional capacity^22,23^ associated with ILD suggest an even worse scenario. It is increasingly recognized that aging and related extrapulmonary manifestations, such as cardiovascular diseases, contribute to morbidity in ILD^8^. In patients with cardiopulmonary disorders, the SSWS is lower than the OWS and is lower than that selected by age- and sex-matched healthy controls. Chronic heart failure patients choose a SSWS with a higher CoT but with lower ventilatory cost^24^. In patients with chronic obstructive pulmonary disease (COPD), the reason for choosing a less economic SSWS seems to be mediated by the balance between lower dyspnea perception and higher gait stability^10^. It is unknown whether similar locomotor reasons exist among patients with ILD. Despite of disease-specific pulmonary differences including respiratory mechanics and the activity of various respiratory muscles, the dyspnea and inspiratory neural drive relationship during exercise are similar between ILD and COPD patients^25,26^.

The aim of this study was to investigate the walking economy and the possible factors influencing SSWS adopted by patients with ILD in comparison to healthy controls. We hypothesized that patients would select their usual walking speed (i.e. SSWS) to achieve a balance between their higher dyspnea and lower gait stability regardless of the most economic walking speed (i.e. OWS).

## Methods

### Subjects and ethics statement

This study included 10 patients with established fibrotic ILD (three with idiopathic pulmonary fibrosis, four with fibrotic non-specific interstitial pneumonia, and three with chronic hypersensitivity pneumonia) and 10 healthy controls matched for age and sex. Respirologists diagnosed ILD based on the patients' clinical characteristics, pulmonary function, high-resolution chest tomography results, and in some cases, lung biopsy results. Patients with an unstable clinical condition who presented with comorbidities such as severe cardiovascular and neuromuscular diseases or musculoskeletal and/or joint injuries that impaired treadmill walking were excluded from the study. Patients on long-term oxygen therapy or those who were unable to complete the tests were also excluded. All participants read and signed an informed consent form. The study protocol was approved by the Ethics Committee of the Hospital de Clínicas de Porto Alegre (150,550).

### Study design

A cross-sectional study was conducted on two different days. On the first day, resting pulmonary function tests and incremental cardiopulmonary exercise tests were performed. On the second day, treadmill walking tests were performed at different speeds to determine CoT and dynamic stability.

### Pulmonary function test

Pulmonary function tests were performed using a computerized system (Jaeger v. 4.31, Jaeger, Wuerzburg, Germany). Forced vital capacity (FVC), forced expiratory volume in the first second (FEV_1_), FEV_1._FVC^−1^ ratio, diffusing capacity of the lung for carbon monoxide (DL_CO_), and total lung capacity (TLC) were recorded and expressed as the percentage of predicted values. All tests were performed according to international standards.

### Cardiopulmonary exercise testing

Cardiopulmonary exercise tests were performed on an ergometric treadmill (General Electric T2100, Fairfield, CA, USA), using a ramp protocol. Participants completed walking familiarization on the treadmill at low speeds and received instructions on the test. They were then fitted with a mask for breath-by-breath gas analyses (Vmax^®^ Encore PFT system, CA, USA), electrodes for ECG (ECG 12-lead CASE system-Cardiosoft, GE Healthcare, Wauwatosa, USA) were placed, and oxygen saturation (SpO_2_) by pulse oximetry was continuously monitored (Nellcor Puritan Bennett—NPB-195, Pleasanton, CA, USA). Patients with ILD and the controls performed different protocols according to their physical fitness to obtain a test duration between 8 and 12 min^27^. The protocol for patients with ILD consisted of a rest period followed by a warm-up of 3 min at 1.5 km h^−1^. The incremental phase began with 2 km h^−1^ and 1% inclination with increments of 0.5 km h^−1^ per minute until the fourth minute. After that, the speed remained constant, and the slope of the treadmill was increased by 2% every minute until volitional exhaustion. In the control group protocol, the warm-up speed was 3 km h^−1^, and the incremental phase began with 4 km h^−1^ and 1% inclination with increments of 0.5 km h^−1^ per minute until the fourth minute. The remaining part of the exercise protocol was similar to that followed by patients with ILD. Dyspnea and leg effort were rated at the end of each stage using the Borg CR-10 scale^28^, and maximal effort was confirmed according to standard physiological and psychological criteria^29^. Peak oxygen uptake (${\dot{\text{V}}}$O_2peak_) was defined as a 20 s average around the highest value during the test^27^.

### Treadmill walking tests

The participants completed 5–10 min of familiarization on the ground and treadmill. The SSWS was first determined as the participants walked across a 15 m hallway three times at a comfortable pace. Times were registered using a stopwatch for a length of 10 m to avoid acceleration and deceleration phases. The reliability was tested extensively using two or three repetitions^30^. The SSWS was calculated by dividing the distance by the time required to cover it. The SSWS tested on the ground was considered as the average value. The SSWS was then tested and determined on a treadmill where a trained evaluator provided instructions for the participants. The treadmill speed indicator panel was covered, so that the participants did not know the speed at which they were walking. Thus, the researchers instructed the participants to walk at their most comfortable speed. The treadmill speed was gradually decreased or increased by 0.5 km h^−1^ several times, until the participants were sure that the speed was their most comfortable walking speed. Only after this confirmation was the treadmill panel uncovered and the SSWS registered^31^. The SSWS was adjusted on the treadmill at a comfortable pace and then four other speeds were defined: two speeds above (+ 20% and + 40%) and two speeds below (− 20% and − 40%) the SSWS. Additionally, all participants walked through an iso-speed of 3.2 km h^−1^. Participants walked at six different speeds for 5 min in a randomized order with the intervals between the trials large enough to return to a heart rate of < 100 bpm. Cardiorespiratory parameters (Vmax® Encore) and SpO_2_ were recorded throughout the data collection period. Dyspnea and fatigue of the lower limbs (Borg CR-10)^26^ were recorded at the end of the 5 min trials. The spatiotemporal variables were continuously recorded using a 2D kinematics technique. A video camera (120 Hz, CASIO Exilim FH25, Tokyo, Japan) was positioned perpendicular to the side of the treadmill. The step phase was defined using video recording cameras and three reflective markers on the fifth metatarsal, calcaneus, and lateral malleolus.

### Data processing

#### Cost of transport, optimal speed, and locomotor rehabilitation index

The average of ${\dot{\text{V}}}$O_2_ (ml kg^−1^ min^−1^) obtained from the last minute of each walking speed was used to calculate CoT^12^. This value was subtracted from the mean standing ${\dot{\text{V}}}$O_2_ and transformed into Joules (J) using the equation: metabolic power = ${\dot{\text{V}}}$O_2_ (4.94 RER + 16.04)/60 (J kg^−1^ s^−1^), where RER was the respiratory exchange ratio^12^. Finally, CoT (J kg^−1^ m^−1^) was calculated as the ratio of metabolic energy (J kg^−1^ s^−1^) and walking speed (m s^−1^). Ventilation minutes ($\dot{\text{V}}$_E_) were divided by the $\dot{\text{V}}$CO_2_ (ventilatory equivalent for carbon dioxide) to determine the ventilatory efficiency^24^.

The OWS (i.e., the walking speed at the minimum CoT) was determined using a second-degree polynomial function:^32^

$$   {\text{OWS}} =  - {\text{b}} \times 2{\text{a}}^{{ - 1}}     $$

where a and b are empirical constants based on CoT as a function of speed from the experimental data obtained from the control and ILD groups, respectively.

The locomotor rehabilitation index represents the similarity of the SSWS to the biomechanical OWS. The biomechanical OWS is calculated as the square root of the product of the Froude number at the OWS (i.e., 0.25), gravitational acceleration (9.81 m s^−2^), and leg length. The locomotor rehabilitation index is the ratio of the SSWS to biomechanical OWS, multiplied by 100%^33^.

### Dynamic stability

The spatiotemporal variables of contact time, swing time, stride frequency, and stride length were obtained by analyzing 10 cycles of the last-minute walking for each speed using the Kinovea^®^ program (0.8.15, Montceau-les-Mines, France). The analysis consisted of determining the frames in which the foot touched the ground and when the foot ceased to have contact with the ground (i.e., unloading), as determined by a trained researcher^34^.

Dynamic stability was calculated using the coefficient of variation (CoV). The stride-to-stride fluctuations were the result of the division between the standard deviation and the mean of the contact time, swing time, stride frequency, and stride length, from at least 10 strides, using a customized algorithm constructed using the LabVIEW software (version 8.5, National Instruments, Austin, TX, USA). The table contains sheets including individual data for all variables in Supplementary Material 01.

### Statistical analyses

Descriptive statistics were used, and data are presented as means and standard deviations. The normal distribution of the data was verified using the Shapiro–Wilk test. Student's *t* tests for independent samples were used to compare pulmonary function and cardiopulmonary exercise test variables between the patients and controls. A two-way repeated-measures ANOVA was used to compare CoT, Borg dyspnea, $\dot{\text{V}}$E/$\dot{\text{V}}$CO_2_, and dynamic stability. The two factors were the group (patient and control) and speed (− 40%, − 20%, SSWS, + 20%, + 40%, and 3.2 km h^−1^). If a significant interaction between the group and speed was found, post hoc t-tests for independent samples and the Bonferroni test were performed. Statistical analyses were performed using the SPSS version 20. The level of significance was set at *p* ≤ 0.05.

## Results

Age, body mass, height, and body mass index did not differ between the groups (*p* > 0.05). The patients showed an average reduction in TLC and DL_CO_ (Table 1). Table 2 shows the cardiorespiratory variables obtained from the cardiopulmonary exercise test. Patients with ILD presented a reduced ${\dot{\text{V}}}$O_2peak_ compared to that of controls (*p* = 0.001), with a tachypneic breathing pattern and significant oxygen desaturation during exercise.

**Table 1:** Participants’ characteristics (Mean ± SD).

Variables	ILD (n = 10)	Control (n = 10)	*p*
Age (years)	63.8 ± 9.2	62.2 ± 8.2	0.685
Gender (n)			
Male	2	2	
Female	8	8	
Body Mass (kg)	65.2 ± 10.6	64.9 ± 12.2	0.248
Height (cm)	156 ± 9	162 ± 8	0.067
BMI (kg m^−2^)	27.1 ± 4.4	24.6 ± 4.4	0.212
**Lung Function**			
FEV_1_. L (%pred)	1.40 ± 0.43 (56 ± 7)	2.49 ± 0.46 (92 ± 16)	**0.001**
FVC. L (%pred)	1.62 ± 0.55 (54 ± 9)	3.13 ± 0.44 (96 ± 13)	**0.001**
FEV_1_/FVC (%pred)	0.87 ± 0.06 (116 ± 16)	0.79 ± 0.09 (107 ± 21)	**0.031**
TLC. L (%pred)	3.29 ± 0.72(74 ± 10)	–	–
DL_CO_. mmol/min/kPa(%pred)	2.64 ± 0.58(38 ± 8)	–	–
SpO_2_%	96.8 ± 2.4	98.5 ± 1.1	0.117

n: number of participants; SD: standard deviation; BMI: body mass index; FVC: forced vital capacity; FEV_1_: forced expiratory volume in one second; TLC = total lung capacity; DL_CO_: diffusing capacity of the lung for carbon monoxide; SpO_2_: oxyhemoglobin saturation by pulse oximetry.

**Table 2:** Measurements during incremental cardiopulmonary exercise testing (Mean ± SD).

Variables	Patients (n = 10)	Controls (n = 10)	*p*
${\dot{\text{V}}}$O_2_ L min^−1^ (%pred)	0.83 ± 0.47 (32 ± 7)	1.94 ± 0.36 (74 ± 10)	**0.001**
${\dot{\text{V}}}$O_2_ mL kg^−1^ min^−1^	12.8 ± 6.90	28.8 ± 3.25	**0.001**
${\dot{\text{V}}}$CO_2_ L min^−1^	0.89 ± 0.52	2.02 ± 0.56	**0.001**
RER	1.21 ± 0.21	1.10 ± 0.08	0.622
$\dot{\text{V}}$E L min^−1^	34 ± 17	61 ± 18	**0.006**
$\dot{\text{V}}$E/MVV	0.69 ± 0.31	0.67 ± 0.19	0.836
*f* breaths min^−1^	48 ± 11	36 ± 5	**0.003**
V_T_	0.74 ± 0.40	1.71 ± 0.35	**0.001**
HR bpm	156 ± 9	158 ± 8	0.957
SpO_2_ final %	82 ± 11	95 ± 1	**0.002**
Delta SPO_2_%	− 15 ± 10	− 3 ± 1	**0.006**
Borg Dyspnea (range)	3–10	0–9	
Borg Leg Effort (range)	0–9	0–10	

${\dot{\text{V}}}$O_2_: oxygen consumption; ${\dot{\text{V}}}$CO_2_: carbon dioxide output; RER: Respiratory Exchange Ratio; $\dot{\text{V}}$E: minute ventilation; MVV: maximum voluntary ventilation; *f*: breathing frequency; V_T_: tidal volume; HR: heart rate; SpO_2_: oxyhemoglobin saturation by pulse oximetry.

ILD patients adopted a significantly lower SSWS compared to controls (2.6 ± 0.9 vs 4.2 ± 0.4 km h^−1^, respectively; *p* = 0.001). OWS and the associated CoT were 3.96 km h^−1^ and 2.91 J kg^−1^ m^−1^, respectively, for ILD patients and 5.06 km h^−1^ and 2.72 J kg^−1^ m^−1^, respectively for controls, while the resulting locomotor rehabilitation index was 53 ± 18% for ILD patients and 84 ± 10% for controls.

**Figure 1:**
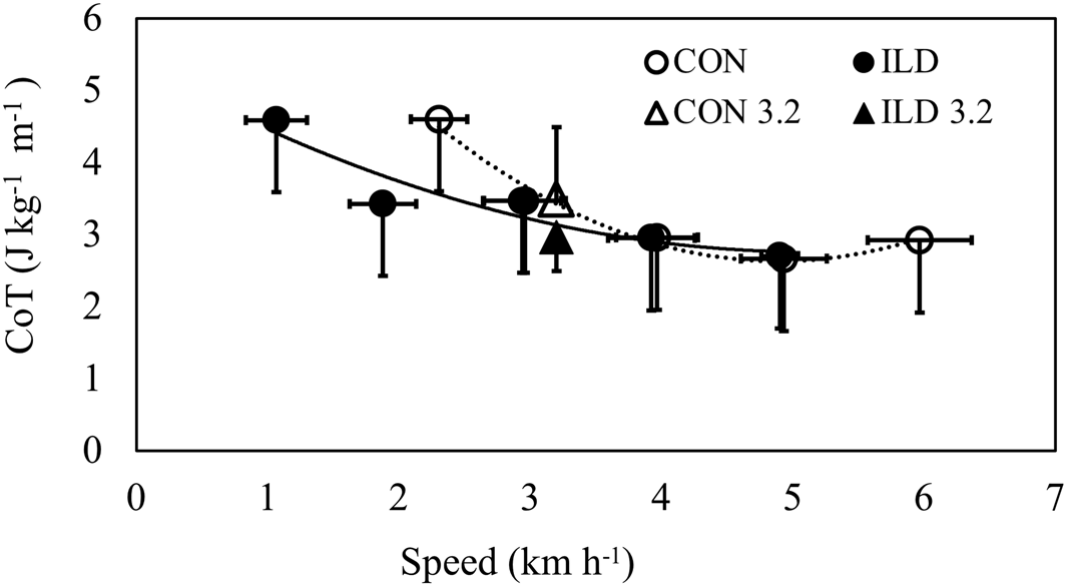
Cost of Transport as a function of absolute speed in patients with interstitial lung disease (ILD, filled circles) and controls (CON, empty circles).

**Figure 2:**
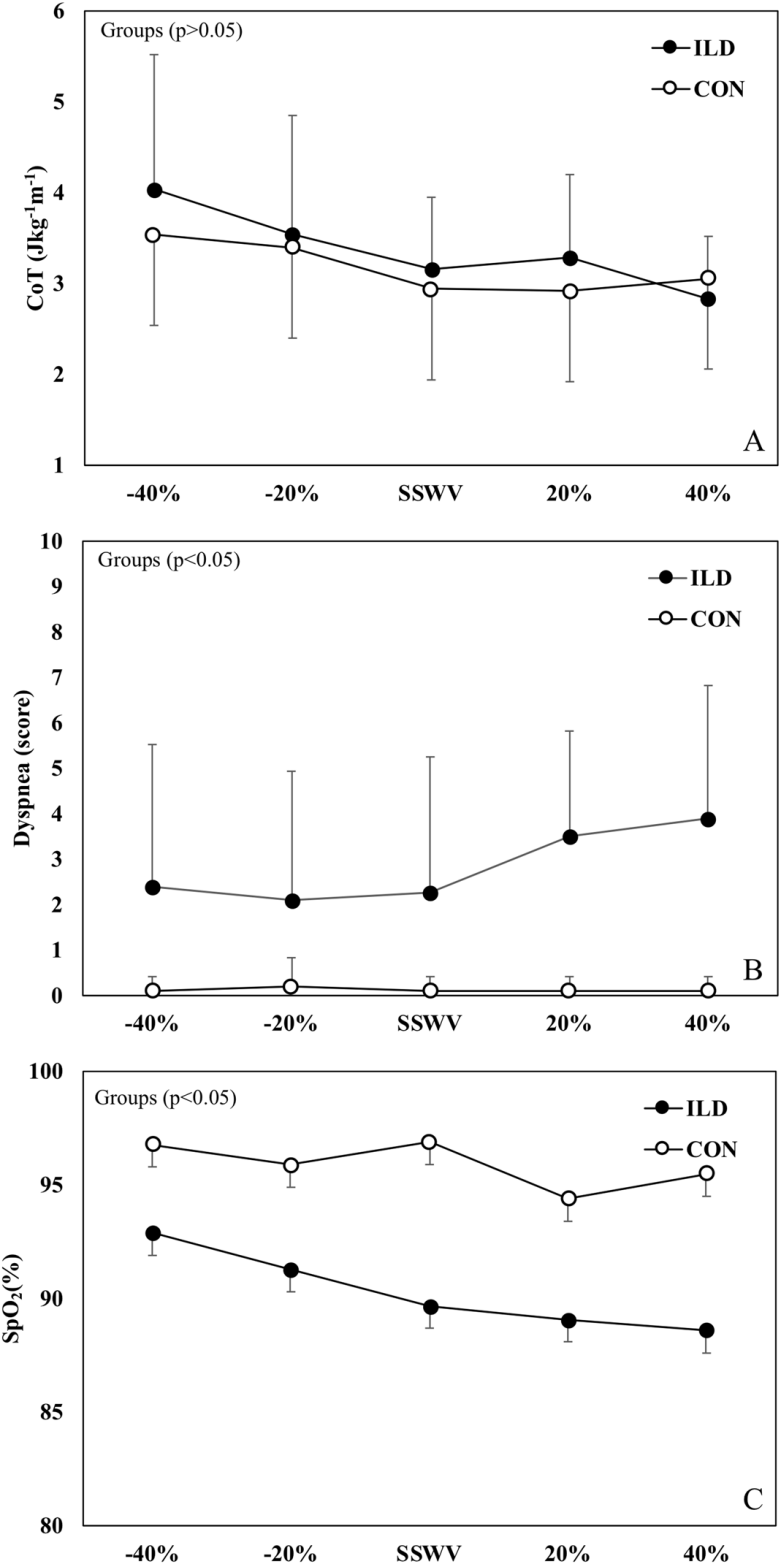
Cost of Transport (CoT; panel **A**), Dyspnea (panel **B**) and Oxyhemoglobin saturation by pulse oximetry (panel **C**) as a function of self-selected walking speed (SSWS), and two relative speeds below and two above SSWS in patients with interstitial lung disease (ILD, filled circles) and controls (CON, empty circles).

Figure 1 shows CoT as a function of the absolute speeds, while Fig. 2 shows CoT, dyspnea, and SpO_2_ as a function of the relative speeds: + 40%, + 20%, SSWS, 20%, and -40%. CoT was not significantly different between the groups (*p* = 0.680) or speeds (*p* = 0.139). No group × speed interaction was observed for CoT (*p* = 0.051). Dyspnea was significantly higher in patients with ILD than in the controls (*p* = 0.005) while SpO_2_ was significantly lower in ILD patients (89.9 ± 2.2% vs. 96 ± 0.8%, respectively; *p* = 0.037). No differences in dyspnea or SpO_2_ were observed at different speeds between the groups (*p* > 0.05).

CoT values did not differ between the groups at iso-speed (i.e., 3.2 km h^−1^), (patients with ILD 3.18 ± 0.9 J kg^−1^ m^−1^ vs. controls 3.48 ± 1.2 J kg^−1^ m^–1^; *p* = 0.555). However, significant between-group differences were found for dyspnea (patients with ILD 4.3 ± 3 vs. controls 0.2 ± 0.6; *p* = 0.002) and SpO_2_ (patients 88 ± 10% vs. controls 97 ± 1.8%; *p* = 0.022) at 3.2 km HP Inc.^−1^. Ventilatory efficiency was influenced by speed (main effect, *p* = 0.008) and group (main effect, *p* < 0.001). Post hoc comparisons showed a reduction in ventilatory efficiency between -40% and + 40% in both groups (*p* = 0.013). Furthermore, no significant interaction was observed (main effect of speed × group, *p* = 0.337). The average values ranged from 45.3 at − 40% speed to 40.7 at + 40% speed for the ILD group, and from 36.1 at − 40% speed to 30.6 at + 40% speed for the control group.

**Figure 3:**
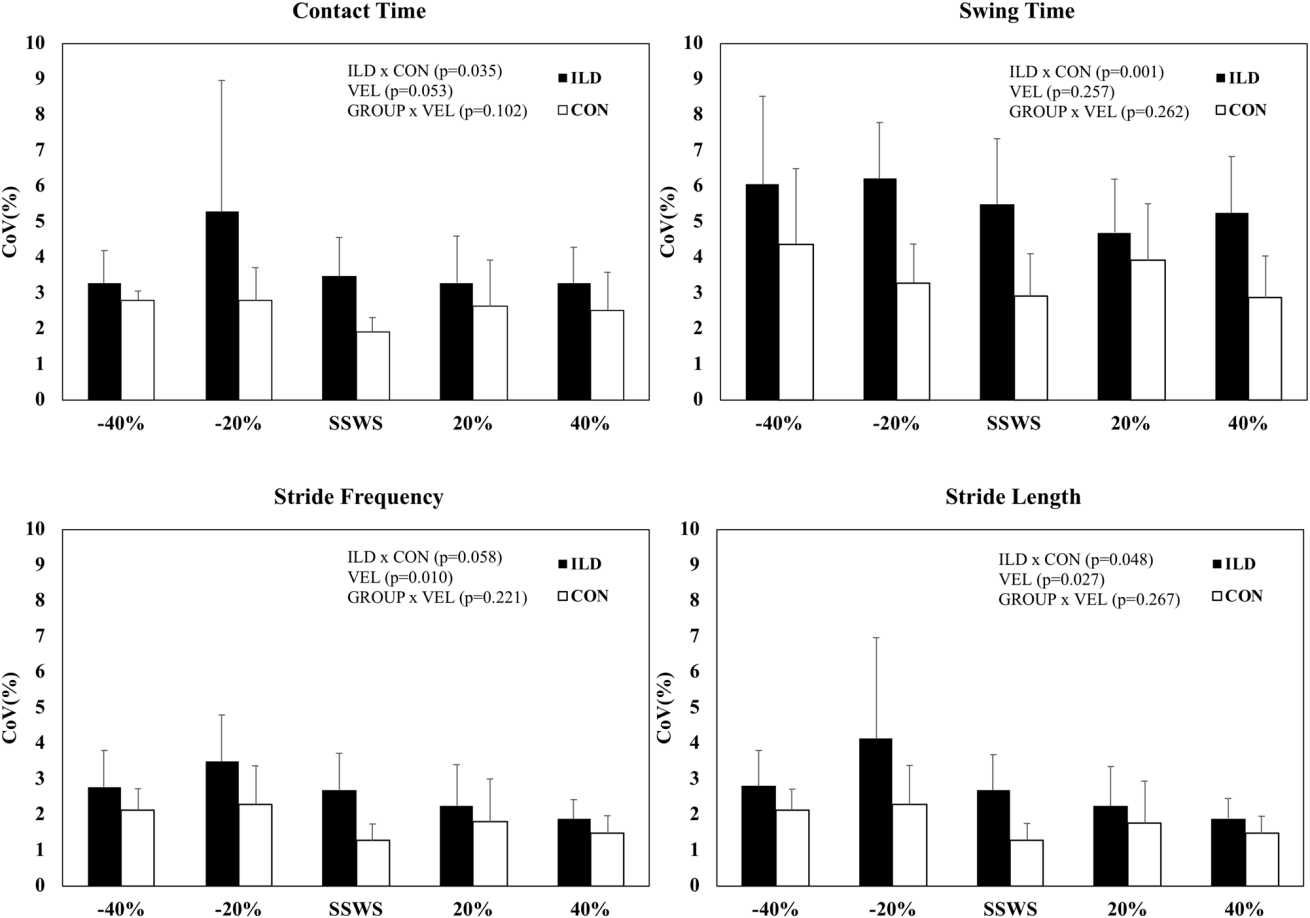
The coefficient of variation (CoV) of contact time, swing time, stride frequency and stride length as a function of self-selected walking speed (SSWS), and two relative speeds below and two above SSWS in patients with interstitial lung disease (ILD, filled columns) and controls (empty columns). The main [group (ILD × CON) and velocity (VEL)] and interaction (GROUP × VEL) effects are denoted.

The CoV for contact time, swing time, and stride length were significantly higher in patients than in controls (Fig. 3), except for stride frequency (*p* = 0.058). CoVs with lower frequency and stride length were observed at higher speeds in the entire sample without interaction group vs. speed.

## Discussion

The present study showed that patients with ILD adopted a lower SSWS than their OWS, in contrast to what was observed for controls. The perception of dyspnea was increased in ILD patients, especially at higher speeds, suggesting that higher breathlessness may discourage the adoption of higher (more energy-efficient) speeds. In contrast, despite worse findings in ILD patients than in controls, parameters such as walking stability and dyspnea did not seem to sufficiently change at different speeds (*p* > 0.05) to a point believed to influence speed selection.

The CoT was not significantly different between the groups and among the speeds. This finding is similar to the results of results of a recent study on patients with COPD^10^. The increased cost of breathing observed in respiratory diseases seems to not be enough to impact the overall metabolic cost of walking^35^. These results support previous findings showing that the walking economy behavior in ILD resembles that observed in obstructive pulmonary disorders^10^. The SSWS of young and healthy people is around 4 km h^−1^ and is believed to be chosen because of the minimum CoT due to optimized pendulum mechanism^13^. Similar findings were observed in the elderly but the minimum CoT values were significantly higher compared to those in the younger individuals^16^. The presence of ILD altered this pattern. The patients' SSWSs were lower than those of the controls and different from their own OWS, which occurred at a higher speed. This means that the patients did not self-select their usual speed with the lowest CoT, as seen in the healthy individuals. The CoT U shape for ILD patients was uncertain because it was not feasible for them to walk at higher speeds (> 5 km h^−1^). It is likely that the trend for an interaction effect group × speed effect (*p* = 0.051) could become significant with extra walking speeds.

Interestingly, based on locomotor rehabilitation index analyses, patients choose to walk at speeds around 50% of the biomechanical optimal speed. Moreover, recent findings showed an improved OWS in older people after Nordic walking training^36^. We speculate that interventions that enhance the speeds that cause moderate or higher dyspnea levels (e.g., Borg scores ≥ 3) would increase the SSWS in individuals with ILD, positively affecting their daily activities.

To our knowledge, this is the first study to investigate dynamic walking stability in patients with ILD. The CoV results of the spatiotemporal variables of walking revealed lower dynamic stability in patients with ILD than in controls. According to Beauchet et al.^37^, because walking is a repetitive movement, its variability does not usually exceed 3%. However, the CoV of the contact time and swing time resulted in mean values above 3% for ILD patients (potentially explained by deteriorated pendulum-like mechanism)^32^. This indicates a higher risk of falls, as observed in patients with COPD^38–40^. Our findings also revealed that walking below or above the SSWS can affect dynamic stability. Indeed, the CoV of stride frequency and length were higher at lower speeds in both the groups. The higher walking variability at low speeds is in line with previous findings of other cardiorespiratory disorders^40^ and stroke patients^34^. It is also worth noting that the stride frequency variability values at SSWS (3%) were higher than those reported by Sanseverino et al. (1.7%) in COPD^10^.

The causes of exertional dyspnea in chronic lung diseases are multifactorial. In particular, dyspnea occurs during exercise under conditions of an abnormal dynamic mechanical/muscular response of the respiratory system and increased central respiratory neural drive^41^. Although the ventilatory and perceptual responses to exercise stress are broadly similar between obstructive and restrictive lung diseases, our findings showed that ILD patients have an additional impairment in the peripheral muscular function (represented here as the dynamic stability) in comparison to obstructive pulmonary disorders^10^. The underlying causes of such differences are uncertain, but could be partly related to the lower cardiorespiratory fitness in ILD patients in the present study (${\dot{\text{V}}}$O_2peak_ = 13 mL kg^−1^ min^−1^) than in Sanseverino et al.^10^ (${\dot{\text{V}}}$O_2peak_ = 19 mL kg^−1^ min^−1^).

Collectively, we speculate that the reduced SSWS is adopted to achieve the combination of the lowest dyspnea sensation (Fig. 2, panel B) despite higher overall gait instability (Fig. 3). Although higher speed would result in a higher economy, patients seem to choose a SSWS lower than the OWS based on lower dyspnea sensation. However, dyspnea was not significantly higher (*p* > 0.05) at higher speeds, probably because of the small sample size. Therefore, these results should be interpreted with caution.

This information may be helpful for physical activity improvement counseling. It may be worth advising patients to perform daily walking activities slightly faster than their SSWS, adopting frequent short pauses before tiring. Despite higher breathing discomfort, walking faster and, therefore, more economically, increases energy conservation and daily physical activity. This strategy needs to be confirmed in future studies using an appropriate randomized controlled trial.

Despite similar CoT, patients with ILD and healthy subjects have different walking strategies. Gait instability and dyspnea perception were significantly higher in patients with ILD. In this context of impaired walking stability, patients seem to adopt a lower SSWS compared to controls and their own OWS, resulting in lower dyspnea perception. From the physiomechanical point-of-view, impairments in gait stability and dyspnea perception are manifestations of disorders in the transmission and muscle efficiencies, respectively^42^. Therefore, ILD patients probably have an overall efficiency lower than controls despite the metabolic economy remaining similar.

## Limitations

The present study had some limitations: the sample size was relatively small, mainly because the experimental design included treadmill tests, and the protocols (integrating biomechanical and physiological procedures) were rather long and wearing. Consequently, some patients could not be included. Walking at higher speeds (> 5 km h^−1^) might have provided a clearer result for the CoT U shape but was not feasible for the ILD patients. In addition, future studies are warranted that use other measures such as the Lyapunov coefficient^43^ and margins of stability^44^ which should provide a deeper understanding of walking dynamical stability. Another limitation was the possible bioenergetic and biomechanical changes that occur during treadmill walking compared to overground walking^45,46^. However, despite these limitations, this study provides a valuable understanding of the SSWS adopted by patients, which could be considered in physical activity programs.

### Ethics statement

This study was carried out in accordance with the recommendations of HCPA Committee with written informed consent from all subjects. All subjects gave written informed consent in accordance with the Declaration of Helsinki. The study protocol was approved by the ethics committee of Hospital de Clínicas de Porto Alegre.

## Supplementary Information

Supplementary Information.
